# Nutritive Evaluation of the Bambara Groundnut Ci12 Landrace [*Vigna subterranea* (L.) Verdc. (*Fabaceae*)] Produced in Côte d’Ivoire

**DOI:** 10.3390/ijms160921428

**Published:** 2015-09-07

**Authors:** Denis N’Dri Yao, Kouakou Nestor Kouassi, Daniela Erba, Francesca Scazzina, Nicoletta Pellegrini, Maria Cristina Casiraghi

**Affiliations:** 1 Laboratory of Food Biochemical and Tropical Products Technology, Nangui Abrogoua University, 02 BP 801 Abidjan, Côte d’Ivoire; E-Mails: ndri_denis@yahoo.fr (D.N.Y.); nestorkksi@yahoo.fr (K.N.K.); 2 Department of Food, Environmental and Nutritional Sciences DEFENS, University of Milan, Via Celoria 2, 20133 Milano, Italy; E-Mails: daniela.erba@unimi.it (D.E.); maria.casiraghi@unimi.it (M.C.C.); 3 Department of Food Science, University of Parma, Parco Area delle Scienze 47/A, 43124 Parma, Italy; E-Mail: francesca.scazzina@unipr.it

**Keywords:** Bambara groundnut (*Vigna subterranea*), nutritional quality, polyphenols, antioxidant capacity, minerals, phytic acid

## Abstract

The nutritional evaluation of the Bambara groundnut Ci12 landrace (*Vigna subterranea* (L.) *Verdc.*) seeds produced in Côte d’Ivoire shows a 19% content of protein, containing all the essential amino acids with tryptophan as the limiting amino acid, a total dietary fiber level of 10%, with a low soluble fraction content, and a fat content of 1.4%, with a high proportion of total unsaturated fatty acids (61%) of which 36% were n-6 fatty acids. This legume contains phosphorus, as the major mineral, followed by magnesium and calcium, and trace elements (iron, copper and zinc). It is characterized by the same amount of α-tocopherol and antioxidant capacity as common legumes. The high concentration of essential amino acids, n-6 fatty acids and minerals, mainly Fe, in the Ci12 landrace of Bambara groundnut indicates that this local legume has the potentiality to improve the nutritional status in Côte d’Ivoire and it could be regarded as a nutrient dense food.

## 1. Introduction

Legume seeds are the most important sources of macronutrients, such as protein, carbohydrates and dietary fiber, in the diet of many populations, especially in developing countries. One of these legumes is the Bambara groundnut, its name is derived from the name of a Mali tribe called “Bambara” [1]. The Bambara groundnut, or round beans, is widespread in Africa where it is known by various names, according to different local language: for instance, among the Akan tribes of Côte d’Ivoire, it is commonly named Clô-Nglô, while in literature the name Bambara groundnut is preferred. This bean is related to cowpeas and it is botanically known as *Vigna subterranea* (L.) Verdc., a member of the *Fabaceae* family.

There are two botanical varieties namely *Vigna subterranea* var. *spontanea*, which includes the wild varieties, and *Vigna subterranea* var. *subterranea*, which includes the cultivated varieties. Although it represents a common food staple in semi-arid area of Africa, the Bambara groundnut remains one of the crops less investigated [2] but one with a great nutritional potential. Unfortunately, the Bambara groundnut has become less important in many parts of Africa because of the expansion of other crop productions. In the recent years, however, there has been renewed interest in such a crop for cultivation in the arid savannah zones. Actually, the Bambara groundnut is known for its resistance to drought and the reasonable yield when grown on poor soils. The Bambara groundnut is the second most important food legume and the third food crop, after maize and groundnut, grown by the small-scale farmers in many African countries. It is also cultivated both as an intercrop with maize, cowpeas and melon and as a sole crop [3].

In all the developing countries, due to the high price of meat and fish, the interest is focused on grain legumes as a source of protein. Legumes are rich not only in proteins, but in other nutrients such as starch and fat [1]. Notwithstanding this, the nutritional value of legume seeds is restricted by the presence of anti-nutrients such as tannin, phytic acid and enzyme inhibitors [1]. Different processing methods such as cooking, roasting and autoclaving, significantly affects the tannin content in Bambara groundnut: in particular, dehulling, soaking and boiling—discarding cooking water—have been shown to be effective in reducing the tannin content [4].

The cultivation of the Bambara groundnut is located in the western and northern areas of Africa characterized by contrasting environments, including tropical rain forests and dry savannas. In these zones, the Bambara groundnut plays a key role in both the diet, used as flour with an improvement of its digestibility [5], and the local culture: for instance, in Côte d’Ivoire, the Bambara groundnut is mainly cultivated by women, and represents a source of income for the household.

Ten landraces of the Bambara groundnut (Ci1, Ci2, Ci3, Ci4, Ci5, Ci7, Ci8, Ci9, Ci10, Ci12) are cultivated according to their areas of origin, their colours and their shapes [6]. However, in order to set up better collecting and conservation strategies their morphological diversities were initially evaluated [7], and only recently agronomic and genetic data have been implemented [6]. Despite the increasing number of scientific reports on the Bambara groundnut in Africa [5,8] only one study [9] is currently available on the overall nutritional quality of seeds from Côte d’Ivoire. Among the available Bambara groundnuts, the Ci12 landrace has a high yield [6] and is the most consumed variety [5] in the north of Côte d’Ivoire. Due to the limited data available in literature on the nutritional quality of the Bambara groundnut, the aim of this study was to investigate the chemical composition and the nutritional potential of this African legume.

## 2. Results and Discussion

The proximate composition, minerals and phytic acid contents of the Bambara groundnut are shown in Table 1. Moisture (11.7 ± 0.1 g/100 g fresh weight, fw) was slightly higher than that reported in literature, a result that could be attributed to its preparation steps and to the environmental condition, while protein and carbohydrate content appears in line with those reported by Falade and Nwajei [8].

**Table 1:** Proximate composition, minerals and phytic acid contents of Bambara groundnut ^a^.

Component	g/100 g fw
Moisture	11.7 ± 0.1
Protein	18.8 ± 0.2
Fat	1.4 ± 0.3
Starch (amylose)	50.2 ± 3.1 17.6 ± 0.5
Sugars	2.4 ± 0.1
Total dietary fiber	10.3 ± 0.0
Soluble fiber	0.5 ± 0.2
Insoluble fiber	9.8 ± 0.2
Ash	2.9 ± 0.0
*Minerals*	
Ca	30.2 ± 1.6
Mg	136.0 ± 2.0
P	335.8 ± 5.9
Fe	8.8 ± 0.6
Cu	0.5 ± 0.0
Zn	1.9 ± 0.1
Phytic Acid	1.1 ± 0.1

^a^ Values are presented as mean ± S.D. (*n* = 3).

In order to assess the nutritional quality of the protein fraction, we evaluated the amino acid content of Bambara groundnut flour and calculated the amino acid score (AAS) (Table 2). The amino acid content is in agreement with that reported by Ihekoronye and Ngoddy [10] showing that the Bambara groundnut is rich in essential amino acids, such as isoleucine, leucine, lysine, methionine, phenylalanine, threonine and valine. In accordance with literature data on African samples [11], glutamic (209.5 mg/g crude protein) and aspartic acids (146.1 mg/g crude protein) are the major non-essential amino acids, while leucine (102.1 mg/g crude protein) and lysine (80.2 mg/g crude protein) are the principal essential amino acids, thus indicating a protein quality very similar to that assessed for different legumes [12]. In accordance with data on *Voadzeiia subterranea* reported by Glew *et al.* [13], the Bambara groundnut Ci12 landrace contains all the essential amino acids, but it does not meet the recommended amino acid patterns specified by FAO/WHO Expert Consultation [14] because of the limited amount of tryptophan. Despite this fact, it could play an important role in meeting the people’s protein needs in combined meals, especially in developing countries.

**Table 2:** Amino acid contents (mean ± S.D., *n* = 3) and amino acid score (AAS) of protein in Bambara groundnut ^a^.

Amino Acid	mg/g Crude Protein	FAO Report mg/g Protein ^a^	AAS
histidine	38.6 ± 2.3	16	2.41
isoleucine	54.5 ± 0.0	30	1.82
leucine	102.1 ± 0.1	61	1.67
lysine	80.2 ± 5.2	48	1.67
threonine	44.3 ± 3.2	25	1.77
tryptophan	6.0 ± 0.0	6.6	0.91
valine	62.4 ± 2.3	40	1.56
methionine	6.4 ± 0.1	-	-
cysteine	24.1 ± 0.0	-	-
SAA	30.5	23	1.33
tyrosine	31.3 ± 1.3	-	-
phenylalanine	76.9 ± 2.0	-	-
AAA	108.2	41	1.88
aspartic acid	146.1 ± 5.1	-	-
serine	68.5 ± 3.5	-	-
glutamic acid	209.5 ± 4.3	-	-
proline	53.6 ± 1.7	-	-
glycine	46.5 ± 3.4	-	-
alanine	51.4 ± 1.4	-	-
arginine	74.8 ± 6.0	-	-
AAS	0.91	-	-
Limiting amino acid	tryptophan	-	-

^a^ Recommended amino acid scoring patterns for older child, adolescence, adult group [14]. SAA: sum of sulphur amino acids (methionine + cysteine); AAA: sum of aromatic amino acids (phenylalanine + tyrosine); AAS: mg of essential amino acid in test protein/mg of essential amino acid in recommended amino acid scoring patterns in FAO report [14].

The fat content was in line with that reported for pulses (Table 1); from a qualitative point of view, the main fatty acids assessed in analyzed Bambara groundnut flour were palmitic (16:0), oleic (18:1 n-9) and linoleic acids (18:2 n-6), representing 21%, 23% and 36% of the total fatty acids content, respectively (Table 3). These data are in agreement with those reported by Minka and Bruneteau [15] for linoleic and palmitic acids but inconsistent for oleic acid, found only in our sample, and linolenic acid found in a great amount in other Bambara seeds (21% *vs.* 1.3% of our sample) as reported by Minka and Bruneteau [15].

**Table 3:** Fatty acid composition (%) of lipid extract from Bambara groundnut ^a^.

Fatty Acids	Common Name	%
16:0	palmitic acid	20.57 ± 0.42
16:1 n-9	palmitoleic acid	0.30 ± 0.05
17:0	margaric acid	0.70 ± 0.02
18:0	stearic acid	7.12 ± 0.18
18:1 n-9	oleic acid	22.61 ± 0.06
18:2 n-6	linoleic acid	35.92 ± 0.17
18:3 n-3	α-linolenic acid	1.30 ± 0.13
20:0	arachidic acid	2.00 ± 0.16
20:1 n-9	gadoleic acid	0.55 ± 0.03
20:2 n-6	*cis*-11.14-eicosadienoic acid	0.07 ± 0.01
20:4 n-6	arachidonic acid	0.05 ± 0.00
22:0	behenic acid	5.41 ± 0.38
24:0	lignoceric acid	1.86 ± 0.16
Total saturated fatty acids	-	37.88
Total monounsaturated fatty acids	-	23.46
Total polyunsaturated fatty acids	-	37.34
Total n-3 fatty acids	-	1.3
Total n-6 fatty acids	-	36.0

^a^ Values are presented as mean ± S.D. (*n* = 3).

The content of carotenoids, tocopherols, total polyphenols and the values of total antioxidant capacity in the analyzed Bambara seeds are shown in Table 4. To the best knowledge of the authors, the content of carotenoids and tocopherols in the Bambara groundnut has not been measured yet. Referring to the results of 30 Brazilian genotypes of cowpea (*Vigna unguiculata* L. Walp) [16] and in agreement with such data, the cowpea analyzed in the present study does not contain a detectable amount of carotenoids. Regarding tocopherols, the only isomer found is α-tocopherol in an amount comparable to common legumes [17], whereas a major amount the δ-isomer has been reported in *Vigna* *unguiculata* L. Walp genotypes [16]. As formerly observed [18], the Trolox equivalent antioxidant capacity (TEAC) value (2.2 mmol of Trolox/100 g) measured by the direct procedure was greater than the ferric reducing antioxidant power (FRAP) one (0.62 mmol of Fe^2+^/100 g) likely due to a low yield of extraction and/or a loss of antioxidants during the hydrolysis treatments applied in this extraction based measurement. However, the values of FRAP and of TEAC were consistent with the data previously determined for common pulses [18,19], even though extremely lower than those recently reported for two varieties of Bambara groundnut [20]. These differences could be likely attributable to several factors, such as different genotype, growing conditions, season, and maturity of the seeds. Nevertheless, the present results demonstrate that the analyzed Bambara groundnut, an underutilized legume, exhibits a comparable antioxidant capacity to commonly consumed legumes, such as chickpea, bean and pea.

**Table 4:** The content of carotenoids, tocopherols, total polyphenols and total antioxidant capacity of Bambara groundnut ^a^.

Component	
Carotenoids	ND
α-Tocopherol (mg/100 g)	0.38 ± 0.09
β-Tocopherol (mg/100 g)	ND
γ-Tocopherol (mg/100 g)	ND
δ-Tocopherol (mg/100 g)	ND
Total polyphenols (mg CE/100 g)	706.9 ± 1.4
FRAP (mmol of Fe^2+^/100 g)	0.62 ± 0.01
TEAC (mmol Trolox/100 g)	2.20 ± 0.01

^a^ Values are presented as mean ± S.D. (*n* = 3); ND: not detectable; CE: catechin equivalents.

Starch is the major and most important energy source in cereals, legumes, and tubers. The structure and functional properties of starch and flour from the Bambara groundnut have been evaluated in different works: the starch granules of the Bambara groundnut are generally more round and smaller than those present in legumes and tend to show a low amylose content for a legume (21.7%) [21]. In the Bambara groundnut Ci12 landrace, we assessed a rough 50% of the nutrients as starch, of which about 35% was amylose (Table 1). This high amylose content could be an interesting trait both in a functional and in a technological point of view. Amylose, in fact, is considered one of the main determinants for a favorable glycemic response [22] and for resistant starch formation, with consequent health benefits on glucose metabolism, energy intake and colonic health [23].

The total dietary fiber content (Table 1) assessed in our samples is almost double when compared to that observed in other Bambara groundnut varieties [8], but appears lower than that reported for legumes such as bean, chickpea, *etc*. The high fiber content evaluated in our samples could probably relate to the thick and hard peel of seeds, not eliminated prior to the flour preparation, or to a stage of over-maturity of the seeds as previously shown for forages [24]. Moreover, the ratio of insoluble to soluble fiber in legumes ranged from 5:1 to 5:19 [25], while, in our Ci12 landrace, it resulted to about 20:1.

The nutritional value of legume seeds is often limited by the presence of anti-nutrients, such as tannins, phytic acid and enzyme inhibitors [1]; tannins reduce the protein digestibility by inhibiting the proteolytic activity and/or by forming indigestible complexes with dietary protein. Our data on total phenol content (707 ± 1.4 mg CE/100 g) was in line with those already reported in the Bambara groundnut [26]. Different studies evidenced that tannins, the principal anti-nutritional fraction of total polyphenols, represent about half of the total phenol content present in these seeds [26] and correlate visually with seed-coat color. However, a recent study shows that kaempferol-3-*O*-glucoside-7-rhamnoside appears to be the most prevalent flavonoid in *V. subterranea* and that, among 11 analysed species of *Vigna*, *V. subterranea* varieties do not contain proanthocyanidins [27], which are known as condensed tannins. Due to the mounting evidence of several beneficial effects towards the human health of these compounds, it would be interesting to investigate, in the future, the specific phenolic compounds present in our samples as well as the best cooking techniques in order to preserve their characteristics.

The content of phytic acid (inositol hexaphosphate) assessed in our samples is higher (1.1 ± 0.1 g/100 g fw) compared to that reported in literature [28]. In several seeds, most of the phosphorus is present as phytic acid and in this structure, due to dissociation of phosphate groups at human intestinal physiological pH, it can bind cations, like calcium, iron or zinc. This binding forms insoluble salts that are not available for absorption, thereby decreasing mineral bioavailability [29]. On the other hand, phytic acid could have several beneficial effects, particularly as a natural antioxidant or as an inhibitor of the cancerous process, despite the fact that the mechanism is still not completely clear [29].

It is now common knowledge that various food-processing methods can reduce and/or destroy most of these anti-nutritional factors. Soaking, cooking, pressure-cooking or germinating induce a variety of physical and biochemical changes in seeds/flours, which could improve their nutritional value [4,26]. In addition, the fermentation, achieved by indigenous microbiota or by the addition of fermented material from a previous production through back slopping, plays an important role in removing anti-nutritional factors. For the Bambara groundnut in particular, isolates from fermenting cotyledons (dawadawa-type product) showed *Bacillus subtilis* and *Bacillus licheniformis* as the major fermenting microorganisms [30]. Some studies emphasized that the fermentation process reduces tannins and trypsin inhibitor activity (by 24% and 40%, respectively) [4] or increases the phenolic content and antioxidant capacity of the fermented Bambara groundnut [31]. Moreover, the fermentation of Bambara seeds has been recently suggested with the aim of producing a vegetable milk, with better sensory properties and suitable as a potential probiotic carrier [1]. In this contest, it would be interesting to carry out “tailored” fermentation processes in order to improve Bambara flours from both nutritional and technological points of view.

The mineral analyses (Table 1) showed that P is the major mineral, as already observed by Amarteifio *et al.* [32], followed by Mg and Ca; their levels (335.8 ± 5.9, 136.0 ± 2.0 and 30.2 ± 1.6 mg/100 g fw, respectively) are generally consistent with those of other varieties grown in Southern Africa, when expressed on dry basis [32]. The Ci12 landrace of Bambara groundnut contains also trace elements like Fe, Cu and Zn (8.8 ± 0.6, 0.5 ± 0.0 and 1.9 ± 0.1 mg/100 g fw, respectively); their contents resulted lower than some varieties of Bambara [11,32], but higher than others [28]. Moreover, considering the amount of phytic acid present in flour, it is conceivable that the bioavailability of these elements, and especially of Fe, is quite low. In this regard, it should be of interest to investigate the effect of the above mentioned processing procedure, such as fermentation, to decrease phytate and thereby improve the potential role of the Bambara flour for producing food items rich in Fe in geographic areas with a high prevalence of iron deficiency.

## 3. Experimental Section

### 3.1. Samples

The Bambara groundnut of the Ci12 landrace (red and grey mottles on cream background, small shape), originated in Sinematialie (north of Côte d’Ivoire), was purchased from several local markets of Abidjan. Samples (*n* = 10) were immediately transferred to the laboratory of Food Biochemical and Tropical Products Technology (Abidjan, Côte d’Ivoire) for the flour preparation. The seeds were cleaned manually to remove all foreign materials, mixed and grinded into fine flour with a laboratory blender (Bimby mod. 2200, Vorwerk, Wuppertal, Germany) prior to analysis. All the analyses were done, in triplicate on flour obtained by pooling all the samples in the same amount, at the Department of Food Science, University of Parma, and at the Department of Food, Environmental and Nutritional Sciences (DeFENS), University of Milan, Italy.

### 3.2. Proximate Composition

The moisture and ash (925.10; 945.38), crude protein (925.31) and lipid (963.15) content were determined in accordance with AACC standard methods [33]. Carbohydrates were evaluated as total starch [34] and simple sugars [35]; the starch fraction was also characterized in terms of amylose content by means of a commercial kit (K-AMYL, Megazyme Int., Wicklow, Ireland). Soluble and insoluble dietary fibre was assessed by the enzymatic gravimetric procedure [36].

### 3.3. Determination of Minerals

The sample was accurately weighted and dry-ashed (550 °C, one night; method 40–70.01) [33] in a muffle furnace (Cavallo Srl, Buccinasco, Italy). Grey ashes were treated with high purity hydrogen peroxide (H_2_O_2_ 30%, Suprapur Merck, Darmstadt, Germany) to obtain white ashes, that were dissolved with acid solution (2 mL HCl 30%, Suprapur Merck) and diluted with distilled water in volumetric flasks. Mineral concentrations (Zn, Fe, Cu, Ca and Mg) were determined by Atomic Absorption (Analyst 800 Perkin Elmer, Waltham, MA, USA), while phosphorous (P) was determined by a colorimetric method using Cary 3E UV-VIS Spectrophotometer (Varian, Mulgrave, Australia) [37]. All analyses were carried out in triplicate and reported as mean and standard deviation; data are expressed as mg/100 g fw.

### 3.4. Determination of Phytic Acid

The phytic acid content was determined by HPLC with post-column sulfosalicylic acid reaction, by using a spectrophotometric detector as described by Oberleas and Harland [38], and quantified in relation to an external standard curve of phytic acid. Before HPLC analysis, suitable aliquots of sample were extracted with 10 mL of 0.66 M HCl for 3 h, centrifuged and filtered (0.45 μm). Results are expressed as g/100 g fw.

### 3.5. Determination of Amino Acids

Five hundred mg of the sample were weighted into a 18 mL Pyrex glass tube fitted with Teflon-lined screw caps and 6 mL of 6 N HCl were added and mixed. The tube was flushed with nitrogen for 1 min in order to remove air. Hydrolysis was then carried out at 110 °C for 23 h. After cooling the tubes at room temperature, the internal standard (7.5 mL of d,l *nor*-leucine 5 mM in water) was added; the mixture was filtered through paper filter and collected into a 250 mL volumetric flask. Acid hydrolysis was used for the determination of all amino acids except tryptophan, cysteine and methionine. For cysteine and methionine performic acid oxidation followed by acid hydrolysis was used. In this case, 500 mg of the sample was weighed in an 18 mL Pyrex glass tube fitted with a Teflon-lined screwcap. After adding 2 mL of neat performic acid freshly prepared, samples were kept in an ice bath for 16 h at 0 °C. Then 0.3 mL of hydrobromic acid was added in order to remove excess performic acid. The bromine formed during the reaction was removed by drying with nitrogen flow. The acid hydrolysis procedure using 6 N HCl as described above was then performed.

In order to prepare a calibration standard solution, 40 μL of d,l *nor*-leucine (2.5 mM in HCl 0.1 N), 40 μL of cysteic acid (2.5 mM in HCl 0.1 N), 40 μL of Amino Acid Hydrolyzate Standard Mixture (EC Number 231-791-2, SIGMA-Aldrich, Stockholm, Sweden) and 880 μL of deionized water were mixed. Then 10 μL of hydrolyzate sample or standard solution were transferred into a 1.5 mL tube, 70 μL of borate buffer were added, in order to keep the optimal pH range for derivatization (8.2–9.7), and the solution was briefly vortexed. Twenty μL of reconstituted AccQ. Fluor reagent was finally added and the mixture was immediately vortexed for several seconds. The tube was closed and left to stand for one minute at room temperature, then heated in a heating bath at 55 °C for 10 min. The resulting derivatized standard solution was diluted with 400 μL of deionized water before injecting in the HPLC system.

The sample and standard solutions were analysed as previously described [39].

### 3.6. Determination of Total Tryptophan by Derivative Spectrophotometry

The sample was prepared by homogenizing 200 mg of the sample with 15 mL NaOH 0.1 N. The sample was centrifuged and the supernatant was collected and diluted 1:5 with NaOH 0.1 N only before the spectrophotometric analysis. The calibration standard solution was prepared with *N*-Acetyl-l-Tryptophanamide 0.2 mM in NaOH 0.1 M (stock solution), then diluted with NaOH 0.1 M to obtain working calibration standard solutions of 0.02, 0.05, 0.08, 0.11, 0.14, 0.17 and 0.20 mM. Quantification of total tryptophan was carried out as previously described by Fletouris *et al.* [40].

### 3.7. Determination of Fatty Acid Composition

The samples were extracted according to the method of Folch *et al.* [41] using a chloroform/methanol mixture in the ratio of 2:1 (*v*/*v*). Methyl esters were prepared from the total lipids by the method of Ackman [42]. Fatty acid methyl esters were analyzed by a Varian 3400 CX gas liquid chromatography equipped with a OMEGAWAX AX 320 column (Supelco, Bellefonte, PA, USA) and a flame ionization detector. Injector and detector temperatures were 250 and 260 °C, respectively. The initial oven temperature was 140 °C and was increased by 2 °C/min to 200 °C and held at this temperature for 25 min; hydrogen at a flow rate of 2.0 mL·min^−1^ was used as carrier gas. A standard fatty acid methyl ester mixture (Omegawax Column test Mix 4-8476 Supelco) was run and retention times were used in identifying the sample peaks; nitrogen at a flow rate of 2.0 mL·min^−1^ was used as carrier gas. A response factor was calculated to correct the GC response of each fatty acid ester to bring them to a common baseline. The methyl ester of pentadecanoic acid (C15:0) was used as an internal standard. Fatty acid levels were estimated on the basis of peak areas of the standards.

### 3.8. Determination of Carotenoids

Carotenoids were extracted according to the method reported by Panfili *et al.* [43], slightly modified as follows: a sample (2 g) was saponified under nitrogen in a screw-capped tube by adding 5 mL of ethanolic butylated hydroxytoluene (BHT, 60 g/L) as antioxidant, 2 mL of ethanol (95% *v*/*v*), 2 mL of sodium chloride (10 g/L), and 2 mL of sodium hydroxide (600 g/L); β-apo-8-carotenal (SIGMA-Aldrich, cod. 10810, Stockholm, Sweden) was added as the internal standard. The tubes were placed in a 70 °C water bath and mixed every 5–10 min. After alkaline digestion at 70 °C for 45 min, the tubes were cooled in an ice bath and samples extracted with 15 mL of THF stabilized with BHT (1 g/L), centrifuged for 5 min at 840× *g*, the supernatant recovered, and the solid material extracted again with 15 mL portions of THF (with BHT). The combined THF extracts were then extracted twice with 40 mL portions of *n*-hexane/ethyl acetate (9:1 *v*/*v*) and 40 mL of NaCl solution (10 g/L). The combined organic layers were collected and evaporated to a dry state and the residue dissolved in 2 mL of methanol: THF (95:5, *v*/*v*). The chromatographic separation of the compounds was achieved by means of a 250 mm × 4.6 mm i.d., 5 μm particle Size C18 polymeric reversed-phase, VYDAC 201TP™ column.

The mobile phase was acetonitrile/methanol/dichloromethane (84:15:1, *v*/*v*) at a flow rate of 1.2 mL/min. Spectrophotometric detection was achieved by a diode array detector (Photodiode array 2996-Waters, Milford, MA, USA) set in the range of 200–600 nm. Carotenoids were identified through their characteristic spectra and retention times in comparison with standards.

### 3.9. Determination of Tocopherols

For this research, 10 g of a sample was saponified with 40 mL KOH (50%, *w*/*v*) plus 120 mL of ethanol (96%, *v*/*v*) in a Soxhlet apparatus for 30 min and then extracted with 100 mL of diethyl ether (adding 10 g of BHT as antioxidant). The organic phase was repeatedly washed with water saturated with ethyl ether to neutral pH and then recovered. The volume was corrected to 250 mL with ethyl ether and 100 mL aliquot evaporated to a dry state; the residue was dissolved in 25 mL of *n*-hexane. HPLC analysis was achieved by means of a 250 mm × 3.3 Lichrosorb Si60 column (Merck) and fluorimetric detection (λ_exc_: 290 nm; λ_em_: 330 nm) with Perkin Elmer LC 24 detector. The mobile phase was *n*-hexane/ethyl acetate (100/7.5, *v*/*v*) at a flow rate of 2 mL/min. Tocopherols were identified through comparison of their retention times with known standard solutions [44].

### 3.10. Determination of Total Antioxidant Capacity

The total antioxidant capacity of the sample was measured by two procedures: (i) a direct procedure based on the TEAC assay and previously described by Açar *et al.* [18]; and (ii) an extraction/hydrolysis procedure. For the latter procedure, the sample was extracted as previously described [19] and analyzed in triplicate for antioxidant capacity by FRAP assay [45]. The antioxidant capacity was expressed as mmol of Trolox equivalents per 100 g fw and mmol of Fe^2+^ equivalents per 100 g fw for the TEAC and FRAP assays, respectively.

### 3.11. Determination of Polyphenols

The phenolic compounds were extracted following the procedure described by Crozier *et al.* [46], and determined by the Folin-Ciocalteu assay using the method described by Adom and Liu [47]. Briefly, the extracts were oxidized with Folin-Ciocalteu reagent, the reaction was neutralized with sodium carbonate and the absorbance was measured at 760 nm. Data were reported as mean ± SD for at least three replications and were expressed as mg catechin equivalents per 100 g fw.

## 4. Conclusions

Our results showed that the Bambara groundnut seed of Ci12 landrace of Côte d’Ivoire is a good source of essential amino acid, n-6 fatty acids and minerals, mainly Fe. With a high yield even when grown on poor soils, the Bambara groundnuts landrace C12 has the potential to improve the nutritional status and could be regarded as a nutrient dense food. Moreover, a tailored fermentation process could be optimized in an attempt to reduce anti-nutrients and, in turn, to improve the bioavailability of minerals and the overall nutritional quality.

## References

[1] Murevanhema Y.Y., Jideani V.A. (2013). Potential of Bambara groundnut (*Vigna subterranea* (L.) Verdc) milk as a probiotic beverage—A review. Crit. Rev. Food Sci. Nutr..

[2] Bamshaiye O.M., Adegbola J.A., Bamishaiye E.I. (2011). Bambara groundnut: An under-utilized nut in Africa. Adv. Agric. Biotechnol..

[3] Swanevelder C.J. (1998). Bambara—Food for Africa.

[4] Barimalaa I.S., Anoghalu S.C. (1997). Effect of processing on certain antinutrients in Bambara groundnut (*Vigna subterranea*) cotyledons. J. Sci. Food Agric..

[5] Hillocks R.J., Bennett C., Mponda O.M. (2012). Bambara nut: A review of utilization, market potential and crop improvement. Afr. Crop Sci. J..

[6] Touré Y., Koné M., Kouakou T.H., Koné D. (2012). Agromorphological and phenological variability of 10 Bambara groundnut [*Vigna subterranea* (L.) Verdc. (Fabaceae)] landraces cultivated in the Ivory Coast. Tropicultura.

[7] Bonny S.B., Djè Y. (2011). Variabilité morphologique et agronomique des variétés traditionnelles de voandzou [*Vigna subterranea* (L.) Verdc. (fabaceae)] de Côte d’Ivoire. J. Appl. Biosci..

[8] Falade K.O., Nwajei C.P. (2015). Physical, proximate, functional and pasting properties of four non- and γ-irradiated Bambara groundnut (*Vigna subterranean*) cultivars. Int. J. Food Sci. Technol..

[9] Djè Y., Béket S.B., Zoro Bi I.A. (2006). Preliminary evaluations on a landrace of Bambara groundnut: Relationships between seed size, chemical composition, germination rate and early seedling growth. Sci. Nat..

[10] Ihekoronye A.I., Ngoddy P.O. (1985). Integrated Food Science and Technology for the Tropics.

[11] Mune M.M.A., Minka S.R., Mbome I.L., Etoa F.X. (2011). Nutritional potential of Bambara bean protein concentrate. Pak. J. Nutr..

[12] Pastor-Cavada E., Juan R., Pastor J.E., Alaiz M., Vioque J. (2014). Protein and amino acid composition of select wild legume species of tribe Fabeae. Food Chem..

[13] Glew R.H., Vander Jagt D.J., Lockett C., Grivetti L.E., Smith G.C., Pastuszyn A., Millson M. (1997). Amino acid, fatty acid, and mineral composition of 24 indigenous plants of Burkina Faso. J. Food Compos. Anal..

[14] FAO/WHO (2013). Dietary Protein Quality Evaluation in Human Nutrition—Report of an FAO Expert Consultation.

[15] Minka S.R., Bruneteau M. (2000). Partial chemical composition of Bambara pea (*Vigna subterranean* (L. *Verde*). Food Chem..

[16] Carvalho A.F.U., de Sousa N.M., Farias D.F., da Rocha-Bezerra L.C.B., da Silva R.M.P., Viana M.P., Gouveia S.T., Sampaio S.S., de Sousa M.B., de Lima G.P.G. (2012). Nutritional ranking of 30 Brazilian genotypes of cowpeas including determination of antioxidant capacity and vitamins. J. Food Compos. Anal..

[17] Kalogeropoulos N., Chiou A., Ioannou M., Karathanos V.T., Hassapidou M., Andrikopoulos N.K. (2010). Nutritional evaluation and bioactive microconstituents (phytosterols, tocopherols, polyphenols, triterpenic acids) in cooked dry legumes usually consumed in the Mediterranean countries. Food Chem..

[18] Açar Ö.Ç., Gökmen V., Pellegrini N., Fogliano V. (2009). Direct evaluation of the total antioxidant capacity of raw and roasted pulses, nuts and seeds. Eur. Food Res. Technol..

[19] Pellegrini N., Serafini M., Salvatore S., del Rio D., Bianchi M., Brighenti F. (2006). Total antioxidant capacity of spices, dried fruits, nuts, pulses, cereals and sweets consumed in Italy assessed by three different *in vitro* assays. Mol. Nutr. Food Res..

[20] Nyau V., Prakash S., Rodrigues J., Farrant J. (2015). Antioxidant activities of Bambara groundnuts as assessed by FRAP and DPPH assays. Am. J. Food Nutr..

[21] Naushad E.M., Taylor J.R.N. (2013). Morphology, physical, chemical, and functional properties of starches from cereals, legumes, and tubers cultivated in Africa: A review. Starch-Starke.

[22] Fitzgerald M.A., Rahman S., Resurreccion A.P., Concepcion J., Daygon V.D., Dipti S.S., Kabir K.A., Klingner B., Morell M.K., Bird A.R. (2011). Identification of a major genetic determinant of glycaemic index in rice. Rice.

[23] Higgins J.A. (2014). Resistant starch and energy balance: Impact on weight loss and maintenance. Crit. Rev. Food Sci. Nutr..

[24] Tassone S., Masoero G., Peiretti P.G. (2014). Vibrational spectroscopy to predict *in vitro* digestibility and the maturity index of different forage crops during the growing cycle and after freeze—Or oven-drying treatment. Anim. Feed Sci. Technol..

[25] Mallillin A.C., Trinidad T.P., Raterta R., Dagbay K., Loyola A.S. (2008). Dietary fibre and fermentability characteristics of root crops and legumes. Br. J. Nutr..

[26] Sowndhararajan K., Siddhuraju P., Manian S. (2011). Antioxidant and free radical scavenging capacity of the underutilized legume, *Vigna vexillata* (L.) A. rich. J. Food Compos. Anal..

[27] Onyilagha J.C., Islam S., Ntamatungiro S. (2009). Comparative phytochemistry of eleven species of *Vigna* (*Fabaceae*). Biochem. Syst. Ecol..

[28] Adegunwa M.O., Adebowale A.A., Bakare H.A., Kalejaiye K.K. (2014). Effects of treatments on the antinutritional factors and functional properties of Bambara groundnut (*Voandzeia*
*subterranea*) flour. J. Food Process. Preserv..

[29] Schelemmer U., Frolich W., Prieto M.R., Grases F. (2009). Phytate in foods and significance for humans: Food sources, intake, processing, bioavailability, protective role and analysis. Mol. Nutr. Food Res..

[30] Barimalaa I.S., Achinewhu S.C., Yibatama I., Amadi E.N. (1994). Studies on the solid substrate fermentation of bambara groundnut (*Vigna subterranea* (L) Verdc). J. Sci. Food Agric..

[31] Ademiluyi A.O., Oboh G. (2011). Antioxidant properties of condiment produced from fermented bambara groundnut (*Vigna subterranea* L Verdc). J. Food Biochem..

[32] Amarteifio J.O., Tibe O., Njogu R.M. (2006). The mineral composition of Bambara groundnut (*Vigna subterranea* (L) Verdc) grown in Southern Africa. Afr. J. Biotechnol..

[33] AACC—American Association of Cereal Chemists (1999). Official Method 40–70.01: Elements by atomic absorption spectrophotometry. Approved Methods of the American Association of Cereal Chemists.

[34] Brighenti F., Casiraghi M.C., Baggio C. (1998). Resistant starch in the Italian diet. Br. J. Nutr..

[35] Zygmunt L.C., Anderson E., Behrens B., Bowers R., Bussey M., Cohen G., Colon M., Deis C., Given P.S., Granade A. (1982). High pressure liquid chromatographic determination of mono-and disaccharides in presweetened cereals: Collaborative study. J. AOAC Int..

[36] Prosky L., Asp N.G., Schweizer T.F., DeVries J.W., Furda I., Lee S.C. (1994). Determination of soluble dietary fiber in foods and food products: Collaborative study. J. AOAC Int..

[37] Fiske C.H., Subbarow Y. (1925). The colorimetric determination of phosphorus. J. Biol. Chem..

[38] Oberleas D., Harland B. (2007). Validation of a column liquid chromatographic method for phytate. J. AOAC Int..

[39] Dall’Asta C., Florio P., Lammardo A.M., Prandi B., Mazzeo T., Budelli A., Pellegrini N. (2015). Development of an *in vitro* digestive model for studying the peptide profile of breast milk. Int. J. Food Sci. Nutr..

[40] Fletouris D.J., Botsoglou N.A., Papageorgiou G.E., Mantis A.J. (1993). Rapid-determination of tryptophan in intact proteins by derivative spectrophotometry. J. AOAC Int..

[41] Folch J., Lees M., Solane-Stanley G.H. (1957). A simple method for the isolation and purification of total lipids from animal tissues. J. Biol. Chem..

[42] Ackman R.G., Hamilton R.G., Rossel J.B. (1986). WCOT (capillary) gas-liquid chromatography. Analysis of Oil and Fats.

[43] Panfili G., Fratianni A., Irano M. (2004). Improved normal-phase high-performance liquid chromatography procedure for the determination of carotenoids in cereals. J. Agric. Food Chem..

[44] Vuilleumier J.P., Keller H.E., Gysel D., Hunziker F. (1983). Clinical chemical methods for the routine assessment of the vitamin status in human populations. Part I: The fat-soluble vitamins A and E, and β-carotene. Int. J. Vitam. Nutr. Res..

[45] Benzie I.F.F., Strain J.J. (1999). Ferric reducing/antioxidant power assay: Direct measure of total antioxidant activity of biological fluids and modified version for simultaneous measurement of total antioxidant power and ascorbic acid concentration. Methods Enzymol..

[46] Crozier A., Lean M.E., McDonald M.S., Black C. (1997). Quantitative analysis of the flavonoid content of commercial tomatoes, onions, lettuce and celery. J. Agric. Food Chem..

[47] Adom K.K., Liu R.H. (2002). Antioxidant activity of grains. J. Agric. Food Chem..

